# Duplex Ultrasound Scan Can Prevent Leg Amputation in Severe Postthrombotic Syndrome: A Case Report

**DOI:** 10.1155/crvm/5555757

**Published:** 2025-03-20

**Authors:** Chien Lin Soh, Carlos Pinho, Manal Ahmad, Alun H. Davies

**Affiliations:** ^1^Academic Section of Vascular Surgery, Department of Surgery and Cancer, Imperial College London, London, UK; ^2^Imperial Vascular Unit, Imperial College Healthcare NHS Trust, London, UK

**Keywords:** case report, duplex ultrasound, postthrombotic syndrome, venous insufficiency, venous ulcers

## Abstract

**Introduction:** Postthrombotic syndrome (PTS) describes a condition arising after an acute deep venous thrombosis (DVT) that is characterised by leg heaviness, discomfort, and recurrent venous ulceration. Venous disease is associated with significant morbidity and impairment of mobility due to pain, infection, and oedema.

**Report:** We present a patient in his 40s attending with left lower limb ulceration, swelling, and refractory pain despite previous best medical management and superficial radiofrequency ablation. A venous duplex ultrasound revealed a trifid femoral vein with a competent and incompetent component. Venography showed patent deep veins but failed to offer the same level of detail as duplex. The patient underwent a femoral vein ligation after multidisciplinary discussion. The role of intraoperative duplex ultrasound was essential. The patient clinically improved and is now free of his venous ulcerations.

**Conclusion:** Duplex provided vital information for surgical planning, which venogram was unable to offer. This is an imaging pitfall that is important to be aware of in patients presenting with recurrent venous disease. Our case highlights the importance of thorough clinical assessment and the value of the Doppler ultrasonography assessment in confirming venous incompetence.

## 1. Introduction

Chronic venous disease (CVD) is a multifactorial condition affecting a significant percentage of the global population [1]. Postthrombotic syndrome (PTS) describes a condition arising after an acute deep venous thrombosis (DVT) that is characterised by leg heaviness, discomfort, and recurrent venous ulceration. Patients may experience a spectrum of symptoms arising from venous hypertension and reflux. PTS is diagnosed with clinical examination and has a significant impact on quality-of-life and healthcare systems. Venous ulceration can arise as a consequence of severe venous insufficiency and is associated with significant morbidity and impairment of mobility [2].

Timely assessment, imaging, and intervention to treat patients are essential. Options for imaging include duplex ultrasonography, computed tomography venogram, magnetic resonance venogram, or endovenous imaging such as contrast venography.

Duplex scanning is often used as first-line noninvasive imaging in an outpatient setting. Contrast venography is an invasive imaging modality that includes the injection of contrast into a vein followed by X-ray. Our case report describes the role and value of duplex ultrasound in evaluating the anatomical variation and direction of the flow, which was not identified on the venogram and aided in operative management. This technique was also proven to be helpful in the guidance of a deep venous intervention, which, in our case, was ligation of the femoral vein.

## 2. Case Report

We present the case of a male patient in his midforties who presents with rapidly progressing lower limb ulceration, swelling, and refractory pain. He reported a left lower limb DVT and pulmonary embolism (PE) prior to his 18^th^ birthday after an inpatient hospital episode. He was discharged on warfarin which was then switched to phenindione and then to rivaroxaban. He remained on this for 15 years and was subsequently diagnosed with antiphospholipid syndrome at which point the rivaroxaban was switched to acenocoumarol. Twenty-six months prior to his presentation to our centre, the patient sustained a leg injury while gardening, which turned into a weeping wound over the proceeding 2 months. He was reviewed by the vascular team at another hospital where he underwent endovenous ablation. The leg ulcer and pain appeared to initially respond to the treatment, but this deteriorated significantly a few weeks later. He was offered compression bandaging to no effect. He was also upset at the prospect of amputation which was offered as a management option.

He attended our unit for a lower limb venous duplex scan. No previous imaging was available for comparison. This initial scan was performed as per the local examination protocol based on the guidelines by the College and Society for Clinical Vascular Science [3–5]. Ideally, patients should be standing for the examination, but the patient had to be scanned in reverse Trendelenburg due to severe pain which allowed visualisation of relevant vessels.

**Figure 1:**
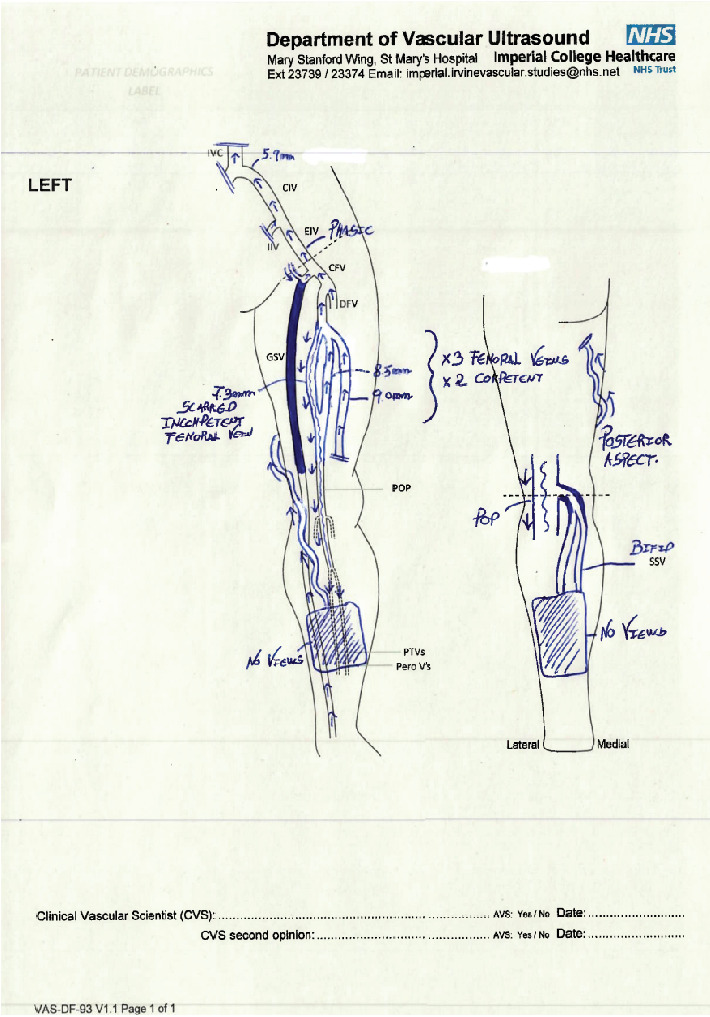
Diagram report for initial/preoperative scan.

The duplex scan highlighted a trifid femoral venous system of which two were competent and the third was scarred and insufficient (> 0.5 s reflux) as demonstrated in Figure 1. The incompetent femoral vein was contributing to the chronic venous insufficiency in the Popliteal vein (Figure 1) patency of the distal inferior vena cava and left iliac veins. The common femoral and proximal deep femoral veins were patent and competent. Three good calibre femoral veins were identified in the upper/midthigh region, and one of these was scarred and grossly incompetent. The popliteal vein was also refluxing and scarred. The saphenofemoral junction appeared patent and competent with normal epigastric flows draining into the common femoral. There was an occlusive thrombus in the midsection of the great saphenous vein suggestive of previous endovenous operative intervention. The distal great saphenous and a tortuous and superficial posteromedial vein appeared competent. These could be the main drainage vessel, but limited views of the lower leg did not allow visualisation of possible incompetent perforators. There was also a nonocclusive thrombus in bifid short saphenous vein.

A diagnostic venogram of his left leg following the duplex reported a patent distal vena cava, left common iliac, external iliac, femoral vein, and proximal popliteal vein. The patient was unable to lie flat in bed due to the pain. The patient refused clinic or emergency department assessment against clinical recommendations.

The patient was discussed at the vascular multidisciplinary team meeting to discuss ligation of the incompetent left femoral vein. The patient was referred for specialist pain team input. A second preoperative venogram revealed a patent distal inferior vena cava, left common iliac, external iliac, femoral vein, and proximal popliteal vein. The profunda confluence was also visualised and was found to be patent. This second venogram did not show any significant difference with the previous study.

The patient was initially prescribed pentoxifylline and a short course of ciprofloxacin as medical management which alleviated the symptoms to some extent. The patient was offered and agreed to operative intervention under general anaesthetic. The left femoral vein (incompetent portion of the trifid) was identified with intraoperative duplex with the assistance of a vascular scientist using a high-resolution probe. This incompetent vein was ligated using a 1/0 Vicryl suture via an anteromedial longitudinal incision under general anaesthetic. Intraoperative Doppler sonography was crucial in confirming the incompetent segment which was not visualised on the venogram.

The patient was reviewed in the clinic 3 months postoperatively, and he was pain and ulcer-free. Once faced with the prospect of major amputation, he had resumed his employment and had a good quality of life. He was grateful to be able to mobilise independently and enjoy an ulcer-free life. The pentoxifylline was stopped at follow-up, and a postoperative duplex scan revealed two good calibre and competent femoral veins. The scarred and incompetent femoral was not visualised as this was ligated. In addition, there was a large incompetent Cockett perforator at the medial ankle draining venous flow into superficial veins that extended to the posterior lower leg and into a competent short saphenous vein.

## 3. Discussion

Patients experiencing chronic venous ulcers arising from venous insufficiency should undergo radiological imaging to confirm the site and extent of the disease and support decision-making for management [6]. Imaging options include the Doppler ultrasound or contrast venography. It is paramount that patients experiencing venous ulceration receive timely treatment, as nonhealing ulcers may place patients at risk of infection and lymphoedema.

Contrast venography is a diagnostic test that is used to directly image the venous system.  Table 1 describes the various imaging findings between different modalities. Both venography scans in our case had false-negative examinations as these failed to identify two competent and one scarred and incompetent femoral veins [7]. As per ESVS guidelines, duplex is recommended as the primary imaging modality [8]. ESVS guidelines highlight variability in patient position, operator dependence, and high over or underdiagnosis rates as limitations of contrast venography [7, 8]. duplex ultrasound is a common primary diagnostic imaging technique that can delineate venous anatomy in patients with chronic venous insufficiency. Despite the difficulty the patient experienced during the duplex ultrasound due to pain, the duplex was able to ascertain the nature of the venous disease and provide a cause of the patient's disease. This was key in the decision-making for surgery which alleviated the patient's symptoms.

It is important to highlight that the patient reported a significant expansion of the ulcer and deterioration in symptoms soon after the great saphenous ablation was performed elsewhere. However, in the author's opinion based on extensive scanning experience, in some patients with severe deep venous damage and venous return via the superficial venous system, it is possible to see reflux lasting for > 0.5 s in the saphenous trunks. Considering that some saphenous trunks may be classed as incompetent while acting as a venous bypass for severe deep vein damage, it may be possible that vascular scientists accurately report reflux in the superficial system, inadvertently misleading surgeons in treatment options. The examination protocol recommends an extensive assessment of the deep and superficial venous systems but does not seem to provide reflux guidance in cases of PTS. [5] Assessment of the venous haemodynamics is paramount in these patients as venous flows may follow unusual paths. It is important to note that this case is unique due to the patient's rare venous anatomy of a trifid femoral vein, which may not be representative of most patients with venous insufficiency.

In conclusion, the preoperative imaging performed at our institution included two invasive venograms and a single noninvasive duplex ultrasound. Clinicians should consider the utility of duplex ultrasound in preoperative planning in patients with severe venous disease.

## Figures and Tables

**Table 1:** Key findings on each imaging modality compared in a table.

**Diagnostic Venogram 1**	**Diagnostic Venogram 2**	**Duplex ultrasound**
• Patent distal vena cava, left common iliac, external iliac, femoral vein, and proximal popliteal vein • Patent profunda confluence	• Similar to diagnostic Venogram 1	• Competent common and profunda femoral veins. • Two good calibre (8.5 and 9.0 mm in diameter) and competent femoral veins. • One good calibre (7.3 mm) scarred and incompetent (> 0.5 s reflux) femoral vein. • Occlusive thrombus in GSV suggesting recent ablation.

## Data Availability

Data is available upon request from the authors.
